# Associations and gastrointestinal symptoms in women with endometriosis in comparison to women with irritable bowel syndrome: a study based on a population cohort

**DOI:** 10.1186/s12876-023-02861-w

**Published:** 2023-07-03

**Authors:** Sofie Stark Junkka, Bodil Ohlsson

**Affiliations:** 1 Department of Clinical Science, Lund University, Malmö, Sweden; 2 Department of Internal Medicine, Skåne University Hospital, Malmö, Sweden

**Keywords:** Endometriosis, Gastrointestinal symptoms, Irritable bowel syndrome, Lifestyle habits, Sociodemographic factors

## Abstract

**Background:**

Endometriosis and irritable bowel syndrome (IBS) have similar symptoms, pathogenesis, and risk factors. These diagnoses often coexist and are frequently misdiagnosed leading to diagnostic delays. This study of a population-based cohort aimed to investigate associations relating to endometriosis and IBS and to compare gastrointestinal symptoms between endometriosis and IBS.

**Method:**

The study cohort included women from the Malmö Offspring Study with information about endometriosis and IBS diagnoses from the National Board of Health and Welfare. The participants answered a questionnaire about lifestyle habits, medical and drug history, and self-reported IBS. The visual analog scale for IBS was used to estimate gastrointestinal symptoms the past 2 weeks. Endometriosis diagnosis and self-reported IBS were used as dependent variables to study associations with age, body mass index (BMI), education, occupation, marital status, smoking, alcohol habits, and physical activity using logistic regression. Mann-Whitney U Test or Kruskal-Wallis tests were used to calculate the differences in symptoms between groups.

**Results:**

Of the 2,200 women with information from medical records, 72 participants had endometriosis; 21 (29.2%) of these had self-reported IBS. Of the 1,915 participants who had answered the questionnaire, 436 (22.8%) had self-reported IBS. Endometriosis was associated with IBS (OR:1.86; 95%CI:1.06–3.26; p = 0.029), as well as with age 50–59 years (OR:6.92; 95%CI:1.97–24.32; p = 0.003), age ≥ 60 years (OR:6.27; 95%CI:1.56–25.17; p = 0.010), sick leave (OR:2.43; 95%CI:1.08–5.48; p = 0.033), and former smoking (OR:3.02; 95%CI:1.19–7.68; p = 0.020). There was an inverse association with BMI (OR:0.36; 95%CI:0.14–4.91; p = 0.031). IBS was associated with endometriosis (OR:1.77; 95%CI:1.02–3.07; p = 0.041) and sick leave (OR:1.77; 95%CI:1.14–2.73; p = 0.010), with a tendency to association with smoking (OR:1.30; 95%CI:0.98–1.72; p = 0.071). When excluding participants using drugs associated with IBS, the condition was associated with current smoking (OR:1.39; 95%CI:1.03–1.89; p = 0.033) and inversely with age 50–59 years (OR:0.58; 95%CI:0.38–0.90; p = 0.015). There were differences in the gastrointestinal symptoms between IBS and healthy participants, but not between endometriosis and IBS or healthy participants.

**Conclusion:**

There were associations between endometriosis and IBS, without differences in gastrointestinal symptoms. Both IBS and endometriosis were associated with smoking and sick leave. Whether the associations reflect causality or depend on common risk factors and pathogenesis remains to be determined.

## Background

Endometriosis is a chronic inflammatory and progressive disease that affects women in their reproductive years [1, 2]. The disease is characterized by endometrial-like tissue outside the uterus [1]. Endometriosis can be found in different locations, most commonly in pelvic structures such as the pelvic peritoneum and the ovary but can also, in unusual cases, be found in the lung, liver, pancreas, and operative scars [3]. The prevalence of endometriosis varies in different studies and depends on diagnostic methods. The prevalence of endometriosis is estimated to be 6–10% in the general population, and 35–50% in women with pain, infertility, or both [4]. The golden standard for endometriosis diagnosis is laparoscopy with histopathological confirmation [3, 5].

Irritable bowel syndrome (IBS) is a disease of the gut-brain interaction (DGBI) with a global prevalence of 1–25% and a pooled prevalence of 3.8%, most frequently found in women [6]. In secondary care, the prevalence of these disorders constitutes 35% of all patients; IBS being the most common [7]. IBS develops no objective findings, making the diagnosis symptom-based using the Rome IV criteria [8].

In both endometriosis and IBS, inflammation with elevated pro-inflammatory cytokine and visceral hypersensitivity are parts of the pathophysiological mechanisms [9–11]. A diet with low fermentable oligo-, di-, monosaccharides and polyols (FODMAP) has been found to have a positive effect on both women with IBS and endometriosis, which supports the thesis of similar pathophysiology [12]. Other similarities are hormonal links [10] and being most common in women under the age of 50 years [13, 14], as well as associations with impaired psychological well-being [15, 16]. Consequently, there is a two- to threefold increased risk for an endometriosis patient to also get the diagnosis of IBS [9, 10, 17]. In a cross-sectional study with endometriosis patients recruited at a tertiary center, the disease was inversely associated with alcohol intake, physical activity, and BMI compared with controls from the general population [18]. On the other hand, self-reported IBS in women from the general population was associated with former smoking [19].

With no simple or cost-effective method to diagnose endometriosis, and since women with endometriosis may fulfill the Rome criteria, endometriosis is often misdiagnosed as IBS [9, 10, 17]. Endometriosis and IBS are treated in different ways, and the delay of the correct diagnosis may lead to unnecessary suffering and a poorer prognosis [9, 10, 17]. Untreated endometriosis may lead to infertility or subfertility [12, 20], while treating IBS with gestagen hormones may increase symptoms of bloating [21]. More knowledge about the character of gastrointestinal symptoms could possibly help women to receive the accurate diagnosis and treatment with a shortened delay. When comparing endometriosis and IBS from two cohorts recruited at a tertiary hospital, IBS patients had more severe gastrointestinal symptoms, except for constipation, and worse psychological well-being [22], and almost half of the endometriosis patients could differ between pain symptoms from endometriosis and from the gastrointestinal tract [23]. Taking all aspects together, the hypothesis was that there could be clinical differences between endometriosis and IBS. The current study with participants from the general population aimed to (1) explore associations of sociodemographic and lifestyle habits between endometriosis and IBS and (2) compare gastrointestinal symptoms and psychological well-being between endometriosis and IBS.

## Methods

### Malmö offspring study – study participants

The Malmö Diet Cancer Study (MDCS) is constituted of 28,098 middle-aged individuals, born between 1923 and 1950, and living in Malmö between 1991 and 1996. From this cohort, subjects were randomly selected for a re-evaluation and constitute the Malmö Diet Cancer Study-Cardiovascular Cohort (MDCS-CC) (n = 6,103). The Malmö Offspring Study (MOS) started in 2013 and included adult children and grandchildren of participants from MDCS-CC. MOS included a web-based questionnaire; dietary registrations; samples from blood, urine, and saliva; cognitive testing; and technical tests, measuring glucose metabolism and cardiovascular- and respiratory function. MOS included a total of 5,277 individuals. In this study, only the web-based questionnaire was used [24].

### Questionnaires

#### Study questionnaire

The MOS web-based questionnaire included questions about living conditions, family connection, education, and profession. Self-perceived health, experience of stress, sleeping habits, medical history, family disease history, medication, and women’s reproductive health were considered. Lifestyle habits in the form of tobacco use, alcohol use, and physical activity were reported [24]. The IBS diagnosis was based on the question “Have you several times a month been bothered by abdominal pain and irregular bowel habits, also called IBS? “ The diagnosis IBS was given if the participants answered yes to the question, which reflects IBS according to the Rome III criteria [25].

#### The visual analog scale for irritable bowel syndrome

The web-based questionnaire asked the question: “Have you experienced any gastrointestinal symptoms in the past 2 weeks?” If the participants answered yes, they were encouraged to continue with the visual analog scale for irritable bowel syndrome (VAS-IBS), which contains seven questions for rating abdominal pain, diarrhea, constipation, bloating and flatulence, vomiting and nausea, intestinal symptoms’ influence on daily life, and psychological well-being. The questions estimate the gastrointestinal symptoms from 0 to 100 mm on a visual analog scale (VAS) where 0 means no and 100 means maximal symptoms. The VAS scales were inverted from the original version [26]. Reference values for healthy women are available [27].

### Diagnosis collection

Information about the included participants’ diagnoses, endometriosis and IBS, was obtained from the National Patient Register, requested from the National Board of Health and Welfare regarding all inpatient care. Information from specialized outpatient care between 1973 and 2000 was requested from the medical records of Region Skåne, and after 2001 information was requested from the National Board of Health and Welfare.

### Data categorization and modification

Age and body mass index (BMI) were not normally distributed and therefore had to be categorized. Age was sorted into < 30, 30–39, 40–49, 50–59, and ≥ 60 years. BMI was classified into < 25, 25–30, and ≥ 30 kg/m^2^ according to the World Health Organization (WHO) standard [28]. Education was grouped into primary school, secondary school, and higher education. The occupation was sorted into working, retired, sick leave, studying, unemployed, and other. Marital status was divided into living alone, living together, and other. Smoking was grouped into never, former, and present smoking. Alcohol habits were sorted based on drinking frequency (≤ 1 time/month, 2–4 times/month, 2–3 times/week, and ≥ 4 times/week) and the amount in glasses per occasion (1–2, 3–4, 5–6, and ≥ 7 glasses). Physical activity at work was grouped into light (sitting or standing), intermediate (walking and lifting < 5 kg), and hard (increased breathing). Physical activity in leisure time was sorted into sedentary (< 120 min per week, without sweating), moderate (move around ≥ 120 min per week, without sweating), moderate but regularly (≥ 30 min per week, sweating), and regularly (≥ 90 min per week, sweating).

The international classification ATC-system (Anatomic Therapeutic Chemical classification system) established by WHO was used for drug categorization [29]. The drugs examined in this study were those previously found to be associated with IBS [30], and included beta-blocking agents (propranolol, metoprolol, atenolol, and bisoprolol), hypnotics and sedatives (zopiclone, zolpidem, and propiomazine), and antihistamines for systemic use (clemastine, alimemazine, promethazine, cetirizine, loratadine, ebastine, fexofenadine, and desloratadine).

### Statistical analysis

The data were analyzed using the statistical software package SPSS, version 28, data for Windows. The variables were not normally distributed and therefore the non-parametric tests Mann-Whitney U test or Kruskal-Wallis test were used to calculate differences between groups. The chi2 test was used for categorical variables. The binary logistic regression model was used to estimate associations. Endometriosis or IBS were used as dependent variables to estimate odds ratios (OR) and 95% confidence intervals (CI) for the independent variables age, BMI, education, occupation, marital status, smoking, drinking frequency, drinking glasses/occasions, and physical activity at work and in leisure time. Adjusted ORs were then calculated with all variables included. A sensitivity analysis was performed where participants who used drugs associated with IBS were excluded [30]. Values are given as numbers and percentages, median and interquartile ranges, or OR and 95% CI. P < 0.05 was considered significant.

## Results

### Study population

**Fig. 1:**
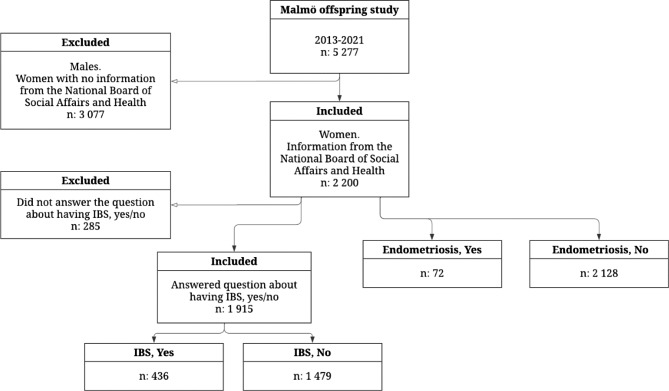
Study population. Flow chart showing the origin of the participants, including excluded individuals and groupings. IBS: irritable bowel syndrome

Of the 5,277 participants enrolled in the MOS, only the 2,200 women with information from the National Board of Health and Welfare were included in this project (Fig. 1). Of the included women, 72 participants had an endometriosis diagnosis, and 61 (84.7%) of them had answered the questions about self-reported IBS. Of these women, 21 (34.4%) had self-reported IBS. Only 22 participants (36.1%) had answered the VAS-IBS questionnaire, and only 2 of them were without any gastrointestinal symptoms at all.

Of the 1,915 participants who had answered the question if they have IBS or not, 436 participants (22.8%) had self-reported IBS. Two of them had not answered the questions about gastrointestinal symptoms. Of the remaining 434 participants, 319 (73.2%) had at least one gastrointestinal symptom (Fig. 1). There was an association between self-reported IBS and IBS diagnoses from the medical records (p < 0.001).

### Endometriosis

The women with endometriosis had a median age of 52.6 years (range 19.6–69.6 years), compared with a median age of 43.3 years (range 18.3–72.9 years) in those without the diagnosis. Participants with endometriosis had a median BMI of 24.6 kg/m^2^ (range 19.0–41.4 kg/m^2^) and those without endometriosis had a median BMI of 24.3 kg/m^2^ (range 15.4–50.1 kg/m^2^).

In the crude calculations, endometriosis was associated with the age groups 40–49 years (OR: 3.60; 95% CI: 1.38–9.55; p = 0.010), 50–59 years (OR: 6.32; 95% CI: 2.63–15.17; p < 0.001), and ≥ 60 years (OR: 5.61; 95% CI: 2.13–14.76; p < 0.001), and sick leave (OR: 2.94; 95% CI: 1.46–5.92; p = 0.002), and inversely associated with moderate (OR: 0.44; 95% CI: 0.20–0.97; p = 0.042) and regularly exerted (OR: 0.35; 95% CI: 0.14–0.89; p = 0.027) physical activity in leisure time (Table 1).

**Table 1:** Characteristics of the endometriosis population

	Endometriosis *N* = 2200					
	**No** 2128 (96.7)	**Yes** 72 (3.3)	**Crude OR** (95% CI)	**P-value** Crude OR	**Adjusted OR** (95% CI)	**P-value** Adjusted OR
**Age (years)**						
<30	618 (29.0)	6 (8.3)	1.00		1.00	
30–39	327 (15.4)	5 (6.9)	1.58 (0.48–5.20)	0.456	1.85 (0.42–8.23)	0.417
40–49	372 (17.5)	13 (18.1)	**3.60 (1.38–9.55)**	**0.010**	4.27 (1.12–16.23)	0.330
50–59	554 (26.0)	34 (47.2)	**6.32 (2.63–15.17)**	**< 0.001**	**6.92 (1.97–24.32)**	**0.003**
≥60	257 (12.1)	14 (19.4)	**5.61 (2.13–14.76)**	**< 0.001**	**6.27 (1.56–25.17)**	**0.010**
**BMI** (kg/m2)						
<25	1189 (55.9)	38 (52.8)	1.00		1.00	
25.0–29.9	574 (27.0)	24 (33.3)	1.31 (0.78–2.20)	0.312	0.79 (0.42–1.49)	0.471
>30	363 (17.1)	10 (13.9)	0.86 (0.42–1.75)	0.68	**0.36 (0.14–0.91)**	**0.031**
Missing	2 (0.1)	0				
**Education**						
Primary school	133 (6.3)	4 (5.6)	1.00		1.00	
Secondary school	1003 (47.1)	36 (50.0)	1.19 (0.42–3.41)	0.741	1.53 (0.42–5.50)	0.517
Higher education	796 (37.4)	21 (29.2)	0.88 (0.30–2.60)	0.813	1.10 (0.29–4.16)	0.889
Missing	196 (9.2)	11 (15.3)				
**Occupation**						
Working	1228 (57.7)	37 (51.4)	1.00		1.00	
Retired	58 (2.7)	2 (2.8)	1.14 (0.27–4.86)	0.855	0.86 (0.17–4.26)	0.853
Sick leave	124 (5.8)	11 (15.3)	**2.94 (1.46–5.92)**	**0.002**	**2.43 (1.08–5.48)**	**0.033**
Studying	196 (9.2)	3 (4.2)	0.51 (0.16–1.66)	0.263	1.58 (0.42–5.95)	0.498
Unemployed	65 (3.1)	1 (1.4)	0.51 (0.63–3.78)	0.510	0.78 (0.10–6.35)	0.813
Other	142 (6.7)	6 (8.3)	1.40 (0.58–3.38)	0.451	2.22 (0.84–5.90)	0.109
Missing/Multiple selections	315 (14.8)	12 (16.7)				
**Marital Status**						
Living alone	514 (24.2)	20 (27.8)	1.00		1.00	
Living together	1260 (59.2)	39 (54.2)	0.80 (0.46–1.38)	0.414	0.74 (0.40–1.35)	0.321
Other	161 (7.6)	1 (1.4)	0.16 (0.02–1.20)	0.074	0.46 (0.05–3.83)	0.469
Missing	193 (9.1)	12 (16.7)				
**Smoking**						
Never	1080 (50.8)	27 (37.5)	1.00		1.00	
Former	323 (15.2)	13 (18.1)	1.61 (0.82–3.16)	0.166	**3.02 (1.19–7.68)**	**0.020**
Present	537 (25.2)	21 (29.2)	1.56 (0.88–2.79)	0.130	1.01 (0.52–1.95)	0.984
Missing	188 (8.8)	11 (15.3)				
**Drinking frequency**						
≤1 time/month	665 (31.3)	22 (30.0)	1.00		1.00	
2–4 times/month	734 (36.8)	24 (33.3)	0.93 (0.52–1.69)	0.799	0.88 (0.45–1.73)	0.714
2–3 times/week	448 (21.1)	14 (19.4)	0.94 (0.48–1.87)	0.870	0.60 (0.28–1.32)	0.207
≥4 times/week	42 (2.0)	1 (1.1)	0.72 (0.1–5.47)	0.751	0.35 (0.04–2.81)	0.321
Missing	190 (8.9)	11 (15.3)				
**Drinking glasses/occasion**						
1–2	1115 (52.4)	45 (62.5)	1.00		1.00	
3–4	445 (20.9)	12 (16.7)	0.67 (0.35–1.28)	0.221	0.77 (0.39–1.54)	0.467
5–6	116 (5.5)	0	0.00 (0.00)	0.996	0.00 (0.00)	0.996
≥7	27 (1.3)	0	0.00 (0.00)	0.998	0.00 (0.00)	0.998
Missing	425 (20.0)	15 (20.8)				
Physical activity work*						
Light	1235 (58.0)	35 (48.6)	1.00		1.00	
Intermediate	168 (7.9)	5 (6.9)	1.05 (0.41–2.72)	0.920	0.72 (0.21–2.47)	0.605
Hard	537 (25.2)	21 (29.2)	1.38 (0.80–2.39)	0.252	-	-
Missing	188 (8.8)	11 (15.3)				
Physical activity leisure time**						
Sedentary	150 (7.0)	9 (12.5)	1.00		1.00	
Moderate	803 (37.7)	21 (29.2)	**0.44 (0.20–0.97)**	**0.042**	0.57 (0.22–1.49)	0.252
Moderate but regularly	546 (25.7)	22 (30.6)	0.67 (0.30–1.49)	0.327	0.98 (0.34-2,65)	0.976
Regularly	434 (20.4)	9 (12.5)	**0.35 (0.14–0.89)**	**0.027**	0.64 (0.21–1.97)	0.439
Missing	195 (9.2)	11 (15.3)				

BMI: body mass index; n = number. The prevalence in each group is presented as numbers and percentages. Logistic regression. Values are given as odds ratio (OR) and 95% confidence interval (CI). Bold values are statistically significant, with a *p*-value < 0.05

*Divided into light (sitting or standing), intermediate (walking and lifting < 5 kg) and hard (increased breading). **Divided into sedentary (< 120 min/week, without sweating), moderate (move around ≥ 120 min/week, without sweating), moderate but regularly (≥ 30 min/week, sweating) and regularly (≥ 90 min/week, sweating)

In the adjusted model, there was a significant association between endometriosis and the age groups 50–59 years (OR: 6.92; 95% CI: 1.97–24.32; p = 0.003) and ≥ 60 years (OR: 6.27; 95% CI: 1.56–25.17; p = 0.010), sick leave (OR: 2.43; 95% CI: 1.08–5.48; p = 0.033), and former smoking (OR: 3.02; 95% CI: 1.19–7.68; p = 0.020). There was also an inverse association between endometriosis and BMI > 30 kg/m^2^ (OR: 0.36; 95% CI: 0.14–0.91; p = 0.031). No significant associations were found between endometriosis and education, marital status, drinking frequency, drinking amount, and physical activity at work and in leisure time in the adjusted model (Table 1).

There was an association between endometriosis and self-reported IBS when adjusted for age, BMI, occupation, and smoking as cofounders (OR: 1.86; 95% CI: 1.06–3.261; p = 0.029).

### Irritable bowel syndrome

The participants with self-reported IBS had a median age of 42.0 years (range 18.5–70.0 years), and the women without self-reported IBS had a median age of 45.4 years (range 18.4–72.9 years). The median BMI of the women with self-reported IBS was 24.2 kg/m^2^ (range 16.6–50.1 kg/m^2^), and those without self-reported IBS had a median BMI of 24.3 kg/m^2^ (range 15.9–50.1 kg/m^2^) (Table 2).

**Table 2:** Characteristics of the IBS population

	IBS *n* = 1915						
	No 1479 (77.2)	Yes 436 (22.8)	Crude OR (95% CI)	P-value	Adjusted OR (95% CI)	P-value Adjusted OR	
**Age (years)**							
<30	374 (25.3)	129 (29.6)	1.00		1.00		
30–39	223 (15.1)	70 (16.1)	0.91 (0.65–1.27)	0.581	0.96 (0.63–1.49)	0.836	
40–49	257 (17.4)	78 (17.9)	0.88 (0.64–1.22)	0.437	0.89 (0.58–1.37)	0.597	
50–59	423 (28.6)	108 (24.8)	**0.74 (0.55–0.99)**	**0.043**	0.72 (0.48–1.07)	0.104	
≥60	202 (13.7)	51 (11.7)	0.73 (0.51–1.06)	0.095	0.71 (0.43–1.19)	0.193	
**BMI** (kg/m2)							
<25	821 (55.5)	246 (56.4)	1.00		1.00		
25.0–29.9	409 (27.7)	115 (26.4)	0.94 (0.73–1.21)	0.620	0.95 (0.71–1.26)	0.702	
>30	249 (16.8)	75 (17.2)	1.00 (0.75–1.35)	0.972	0.83 (0.58–1.18)	0.288	
Missing	0	0					
**Education**							
Primary school	100 (6.8)	29 (6.7)	1.00		1.00		
Secondary school	743 (50.2)	234 (53.7)	1.09 (0.70–1.68)	0.712	1.12 (0.66–1.89)	0.671	
Higher education	630 (42.6)	170 (39.0)	0.93 (0.60–1.46)	0.752	1.10 (0.64–1.90)	0.729	
Missing	6 (0.4)	3 (0.7)					
**Occupation**							
Working	998 (67.5)	262 (60.1)	1.00		1.00		
Retired	50 (3.4)	10 (2.3)	0.76 (0.38–1.52)	0.441	0.95 (0.44–2.04)	0.891	
Sick leave	90 (6.1)	45 (10.3)	**1.90 (1.30–2.79)**	**< 0.001**	**1.77 (1.14–2.73)**	**0.010**	
Studying	147 (9.9)	51 (11.7)	1.32 (0.94–1.87)	0.115	1.10 (0.72–1.68)	0.646	
Unemployed	49 (3.3)	17 (3.9)	1.32 (0.75–2.33)	0.336	1.20 (0.63–2.28)	0.581	
Other	113 (7.6)	34 (7.8)	1.15 (0.74–1.72)	0.511	0.99 (0.61–1.59)	0.960	
Missing/Multiple selections	32 (2.2)	17 (3.9)					
**Marital Status**							
Living alone	369 (24.9)	125 (28.7)	1.00		1.00		
Living together	984 (66.5)	268 (61.5)	0.80 (0.63–1.03)	0.079	0.83 (0.63–1.09)	0.176	
Other	120 (8.1)	42 (9.6)	1.03 (0.69–1.55)	0.875	0.77 (0.46–1.27)	0.304	
Missing	6 (0.4)	1 (0.2)					
**Smoking**							
Never	844 (57.1)	226 (51.8)	1.00		1.00		
Former	218 (14.7)	81 (18.6)	**1.39 (1.03–1.86)**	**0.029**	1.24 (0.80–2.06)	0.300	
Present	416 (28.1)	129 (29.6)	1.16 (0.90–1.48)	0.243	1.30 (0.98–1.72)	0.071	
Missing	1 (0.1)	0					
**Drinking frequency**							
≤1 time/month	474 (32.0)	173 (39.7)	1.00		1.00		
2–4 times/month	609 (41.2)	161 (36.9)	**0.72 (0.57–0.93)**	**0.010**	0.80 (0.60–1.06)	0.119	
2–3 times/week	362 (24.5)	90 (20.6)	**0.68 (0.51–0.91)**	**0.009**	0.82 (0.58–1.15)	0.246	
≥4 times/week	32 (2.2)	11 (2.5)	0.94 (0.46–1.91)	0.868	0.99 (0.46–2.13)	0.979	
Missing	2 (0.1)	1 (0.2)					
**Drinking glasses/occasion**							
1–2	896 (60.6)	259 (59.4)	1.00		1.00		
3–4	362 (24.4)	94 (21.6)	0.90 (0.69–1.17)	0.429	0.82 (0.62–1.10)	0.183	
5–6	89 (6.0)	26 (6.0)	1.01 (0.64–1.60)	0.964	0.89 (0.54–1.48)	0.663	
≥7	17 (1.2)	10 (2.3)	2.04 (0.92–4.50)	0.079	1.68 (0.72–3.92)	0.231	
Missing	115 (7.8)	47 (10.8)					
Physical activity work*							
Light	951 (64.3)	264 (60.6)	1.00		1.00		
Intermediate	111 (7.5)	43 (9.9)	1.40 (0.96–2.04)	0.084	1.022 (0.58–1.81)	0.940	
Hard	416 (28.1)	129 (29.6)	1.12 (0.88–1.42)	0.366	-		
Missing	1 (0.1)	0					
Physical activity leisure time**							
Sedentary	106 (7.2)	44 (10.1)	1.00		1.00		
Moderate	594 (40.2)	186 (42.7)	0.75 (0.51–1.11)	0.155	0.86 (0.55–1.35)	0.514	
Moderate but regularly	433 (29.3)	113 (25.9)	**0.63 (0.42–0.94)**	**0.026**	0.73 (0.45–1.19)	0.207	
Regularly	340 (23.0)	91 (20.9)	**0.64 (0.42–0.98)**	**0.041**	0.72 (0.42–1.18)	0.189	
Missing	6 (0.4)	2 (0.5)					

BMI: body mass index; IBS = irritable bowel syndrome; n = number. The prevalence in each group is presented as numbers and percentages. Logistic regression. Values are given as odds ratio (OR) and 95% confidence interval (CI). Bold values are statistically significant, with a *p*-value < 0.05

*Divided into light (sitting or standing), intermediate (walking and lifting < 5 kg) and hard (increased breading). **Divided into sedentary (< 120 min/week, without sweating), moderate (move around ≥ 120 min/week, without sweating), moderate but regularly (≥ 30 min/week, sweating) and regularly (≥ 90 min/week, sweating)

In crude calculations, self-reported IBS was associated with sick leave (OR: 1.90; 95% CI: 1.30–2.79; p < 0.001) and former smoking (OR: 1.39; 95% CI: 1.03–1.86; p = 0.029), and inversely associated with the ages 50–59 years (OR: 0.74; 95% CI: 0.53–0.99; p = 0.043), a drinking frequency of 2–4 times/month (OR: 0.72; 95% CI: 0.57–0.93; p = 0.010) and 2–3 times/week (OR: 0.68; 95% CI: 0.51–0.92; p = 0.009), and moderate but regularly (OR: 0.63; 95% CI: 0.42–0.94; p = 0.026) and regularly exerted (OR: 0.64; 95% CI: 0.42–0.98; p = 0.041) physical activity in leisure time (Table 2).

After adjustment for all variables, there was a significant association between self-reported IBS and sick leave (OR: 1.77; 95% CI: 1.14–2.73; p = 0.010), and a tendency to association with present smoking (OR: 1.30; 95% CI: 0.98–1.72; p = 0.071) (Table 2).

There was an association between self-reported IBS and endometriosis when adjusted for occupation and smoking (OR: 1.77; 95% CI: 1.02–3.07; p = 0.041).

In the sensitivity analysis, self-reported IBS was inversely associated with the age groups 50–59 years (OR: 0.62; 95% CI: 0.45–0.85; p = 0.003) and ≥ 60 years (OR: 0.65; 95% CI: 0.43–0.98; p = 0.040), and with a drinking frequency of 2–4 times/month (OR: 0.73; 95% CI: 0.56–0.95; p = 0.021) and 2–3 times/week (OR: 0.72; 95% CI: 0.52–0.98; p = 0.038). After adjustment, self-reported IBS was inversely associated with the ages 50–59 years (OR: 0.58; 95% CI: 0.38–0.90; p = 0.015) and positively associated with present smoking (OR: 1.39; 95% CI: 1.03–1.89; p = 0.033) (Table 3). No significant associations were found between self-reported IBS and BMI, education, marital status, drinking frequency, drinking amount, and physical activity at work and in leisure time after adjustment for confounders (Tables 2 and 3).

**Table 3:** IBS population with drug use participants excluded

	IBS *n* = 1699						
	No 1337 (76.7)	Yes 362 (21.3)	Crude OR (95% CI)	P-value	Adjusted OR (95% CI)	P-value Adjusted OR	
**Age (years)**							
<30	355 (26.6)	122 (33.7)	1.00		1.00		
30–39	212 (15.9)	62 (17.1)	0.85 (0.60–1.21)	0.366	0.86 (0.55–1.34)	0.496	
40–49	237 (17.7)	63 (17.4)	0.77 (0.55–1.09)	0.145	0.77 (0.49–1.21)	0.260	
50–59	371 (27.7)	79 (21.8)	**0.62 (0.45–0.85)**	**0.003**	**0.58 (0.38–0.90)**	**0.015**	
≥60	162 (12.1)	36 (9.9)	**0.65 (0.43–0.98)**	**0.040**	0.68 (0.39–1.19)	0.179	
**BMI** (kg/m2)							
<25	767 (57.4)	213 (58.8)	1.00		1.00		
25.0–29.9	353 (26.4)	95 (26.6)	0.97 (0.74–1.27)	0.821	1.01 (0.75–1.38)	0.930	
>30	217 (16.2)	54 (14.9)	0.90 (0.64–1.25)	0.520	0.83 (0.56–1.23)	0.360	
Missing	0	0					
**Education**							
Primary school	88 (6.6)	20 (5.5)	1.00		1.00		
Secondary school	669 (50.0)	199 (55.0)	1.31 (0.78–2.18)	0.302	1.48 (0.80–2.73)	0.208	
Higher education	575 (43.0)	140 (38.7)	1.07 (0.64–1.80)	0.795	1.35 (0.72–2.54)	0.350	
Missing	5 (0.4)	3 (0.8)					
**Occupation**							
Working	905 (67.5)	262 (62.2)	1.00		1.00		
Retired	40 (3.0)	10 (1.9)	0.70 (0.31–1.59)	0.399	0.85 (0.35–2.08)	0.728	
Sick leave	77 (5.8)	45 (6.4)	1.20 (0.74–1.96)	0.461	1.25 (0.73–2.14)	0.411	
Studying	136 (10.2)	51 (13.3)	1.42 (0.99–2.04)	0.056	1.11 (0.72–1.72)	0.631	
Unemployed	49 (3.7)	17 (3.9)	1.15 (0.62–2.12)	0.656	1.01 (0.50–2.02)	0.978	
Other	103 (7.7)	34 (8.0)	1.13 (0.73–1.75)	0.577	0.92 (0.55–1.54)	0.755	
Missing	27 (2.0)	16 (4.4)					
**Marital Status**							
Living alone	331 (24.8)	94 (26.0)	1.00		1.00		
Living together	886 (66.3)	228 (63.0)	0.91 (0.69–1.19)	0.477	0.91 (0.67–1.23)	0.542	
Other	115 (8.6)	39 (10.8)	1.19 (0.78–1.84)	0.418	0.93 (0.55–1.57)	0.783	
Missing	5 (0.4)	1 (0.3)					
**Smoking**							
Never	772 (57.7)	196 (54.1)	1.00		1.00		
Former	200 (15.0)	56 (15.5)	1.10 (0.79–1.54)	0.537	0.89 (0.50–1.58)	0.688	
Present	364 (27.2)	110 (30.4)	1.19 (0.91–1.55)	0.197	**1.39 (1.03–1.89)**	**0.033**	
Missing	1 (0.1)	0					
**Drinking frequency**							
≤1 time/month	432 (32.3)	143 (39.5)	1.00		1.00		
2–4 times/month	551 (41.2)	133 (36.7)	**0.73 (0.56–0.95)**	**0.021**	0.77 (0.57–1.05)	0.100	
2–3 times/week	324 (24.2)	77 (21.3)	**0.72 (0.52–0.98)**	**0.038**	0.86 (0.60–1.25)	0.434	
≥4 times/week	28 (2.1)	8 (2.2)	0.86 (0.38–1.94)	0.721	0.95 (0.39–2.31	0.916	
Missing	2 (0.1)	1 (0.3)					
**Drinking glasses/occasion**							
1–2	795 (59.5)	213 (58.8)	1.00		1.00		
3–4	338 (25.3)	83 (22.9)	0.92 (0.69–1.22)	0.547	0.83 (0.61–1.13)	0.231	
5–6	86 (6.4)	22 (6.1)	0.96 (0.58–1.56)	0.854	0.86 (0.50–1.48)	0.588	
≥ 7	16 (1.2)	8 (2.2)	1.87 (0.79–4.42)	0.156	1.47 (0.59–3.71)	0.410	
Missing	102 (7.6)	36 (9.9)					
Physical activity work*							
Light	866 (64.8)	216 (59.7)	1.00		1.00		
Intermediate	106 (7.9)	36 (9.9)	1.36 (0.91–2.04)	0.137	1.42 (0.73–2.78)	0.305	
Hard	364 (27.2)	110 (30.4)	1.21 (0.93–1.57)	0.148	-		
Missing	1 (0.1)	0					
Physical activity leisure time**							
Sedentary	96 (7.2)	30 (8.3)	1.00		1.00		
Moderate	525 (39.3)	148 (40.9)	0.90 (0.58–1.41)	0653	0.98 (0.58–1.65)	0.935	
Moderate but regularly	397 (29.7)	101 (27.9)	0.81 (0.51–1.30)	0.386	0.87 (0.51–1.50)	0.617	
Regularly	313 (23.4)	81 (22.4)	0.83 (0.51–1.34)	0.439	0.82 (0.47–1.44)	0.494	
Missing	6 (0.4)	2 (0.6)					

BMI: body mass index; IBS = irritable bowel syndrome; n = number. Sensitivity analysis, with participants using drugs associated with IBS excluded [30]. The prevalence in each group is presented as numbers and percentages. Logistic regression. Values are given as odds ratio (OR) and 95% confidence interval (CI). Bold values are statistically significant, with a *p*-value < 0.05

*Divided into light (sitting or standing), intermediate (walking and lifting < 5 kg) and hard (increased breading). **Divided into sedentary (< 120 min/week, without sweating), moderate (move around ≥ 120 min/week, without sweating), moderate but regularly (≥ 30 min/week, sweating) and regularly (≥ 90 min/week, sweating)

### Gastrointestinal symptoms

A comparison of the gastrointestinal symptoms was made between women with endometriosis, women with self-reported IBS, women with both endometriosis and self-reported IBS, and healthy women. There was a significant difference in pain, diarrhea, constipation, bloating/gases, intestinal symptoms’ influence on daily life, and psychological well-being (p < 0.001) between groups, with a tendency to differences in vomiting and nausea (p = 0.051). Significant differences were observed between healthy participants and self-reported IBS regarding abdominal pain, diarrhea, constipation, intestinal symptoms’ influence on daily life, and psychological well-being (p < 0.001 for all), and vomiting and nausea (p = 0.016). There was also a significant difference in abdominal pain (p = 0.004), constipation (p = 0.018), bloating/gases (p = 0.026), intestinal symptoms’ influence on daily life (p = 0.008), and psychological well-being (p = 0.002) between healthy vs. endometriosis and self-reported IBS (Table 4). There were no differences between endometriosis and IBS regarding abdominal pain (p = 0.116), diarrhea (p = 0.320), constipation (p = 0.306), bloating and flatulence (p = 0.395), vomiting and nausea (p = 0.219), the intestinal symptoms’ influence on daily life (p = 0.527), and psychological well-being (p = 0.092).

**Table 4:** Gastrointestinal symptoms of the study population

	Endometriosis		P- value	IBS		P-value	Endometriosis + IBS		P-value	Healthy participants*		P-value*
	Median (IQR)	No		Median (IQR)	No		Median (IQR)	No		Median (IQR)	No	
**Pain** 5 (1–15)	26 (4–56)	8	0.628	50 (22–67)	280	**< 0.001**	58 (25–70)	11	**0.004**	20 (4–48)	193	**< 0.001**
**Diarrhea** 3 (0–10)	19 (4–44)	7	0.480	40 (10–67)	272	**< 0.001**	44 (6–56)	10	0.147	10 (1–48)	193	**< 0.001**
**Constipation** 9 (1–22)	29 (1–50)	7	0.471	47 (5–69)	264	**< 0.001**	45 (21–51)	11	**0.018**	9 (0–47)	193	**< 0.001**
**Bloating/flatulence** 13 (1–29)	40 (3–84)	9	0.475	66 (48–79)	283	**< 0.001**	67 (25–74)	13	**0.026**	31 (10–60)	197	**< 0.001**
**Vomiting/nausea** 2 (0–3)	1 (0.-29)	7	0.366	18 (0–54)	258	**0.016**	18 (3–61)	11	0.167	4 (0–48)	189	0.051
**Symptoms´** **influence on daily life** 4 (0–16)	50 (7–76)	9	0.185	58 (24–75)	287	**< 0.001**	50 (25–65)	13	**0.008**	21 (5–50)	202	**< 0.001**
**Psychological well-being** 2 (0–18)	20 (6–46)	36	0.579	30 (14–58)	388	**< 0.001**	49 (23–60)	19	**0.002**	20 (7–35)	1331	**< 0.001**

Symptoms in the past 2 weeks were measured by the visual analog scale for irritable bowel syndrome (VAS-IBS) where 0 mm means no symptoms and 100 mm means maximal symptoms [26]. Reference values for healthy women are given within brackets [27]. Values are presented as the median and interquartile range (IQR). Mann-Whitney U test was used for comparison with healthy participants and Kruskal-Wallis test* at group level. P-value < 0.05 was considered statistically significant

* Participants without endometriosis and/or IBS

## Discussion

The main findings of the present study based on a population cohort were that endometriosis and IBS were associated with each other. Endometriosis was associated with higher age, sick leave, and former smoking, whereas there was an inverse association with BMI. Further, self-reported IBS was associated with sick leave, with a tendency towards an association with present smoking. When excluding participants using drugs associated with IBS [30], self-reported IBS was inversely associated with age and positively associated with smoking. There was a difference in gastrointestinal symptoms between women with IBS and healthy participants, but no significant differences were found among women with endometriosis compared to women with IBS or healthy participants.

Endometriosis is diagnosed at all levels of health care [31]. This project, however, only used information from specialized inpatient and outpatient care, which might have resulted in the lower prevalence of endometriosis compared to other studies [4], with a slightly higher age [13].

The observed association between endometriosis and IBS is in line with three systematic reviews which all observed a two- to threefold risk of getting an IBS diagnosis if the women had endometriosis [9, 10, 17]. In two reviews an increased risk was observed to get an endometriosis diagnosis if the women also had a history of IBS [9, 17]. When no organic changes are found in patients complaining of gastrointestinal symptoms, the IBS diagnosis is used. Due to similar symptoms, the diagnostic delay of endometriosis may be up to 7 years [32]. To resolve this problem, a new algorithm for endometriosis diagnosis has recently been developed and published [33].

The associations noted between the two diseases may be casual or may be based on sharing the same background encompassing chronic inflammatory visceral hypersensitivity [9, 10]. Numerous endometriosis patients have undergone surgical procedures, as could also be the truth for patients with diffuse abdominal pain later called IBS. Laparoscopic procedures or other surgery may in some cases lead to development of visceral hypersensitivity, further triggering pain experience and obscuring the difference between endometriosis and IBS [34]. The association between endometriosis and IBS may also exist due to their common risk factors; being a woman under the age of 50 years [6, 13, 14].

Around half of the endometriosis patients could differentiate between symptoms from endometriosis and symptoms from the gastrointestinal tract [23]. The median duration of gastrointestinal symptoms was 5 months shorter than for other symptoms of endometriosis, although the gastrointestinal symptoms may be the first symptoms in some endometriosis patients. Several cases had received the IBS diagnosis before endometriosis [17, 23]. If young women present with varying symptoms from internal organs, the physician´s specialty has great impact on which diagnosis is given, since the training in considering other options than the own field is limited.

An inverse association between endometriosis and BMI was observed, which is in line with previous research [18, 35]. Association between endometriosis and sick leave and smoking are in line with a larger study cohort with selected endometriosis patients, where the same tendency was observed although not statistically significant [18]. In contrast, others have not observed associations between endometriosis and smoking [36].

Endometriosis is thought to be an inflammatory-driven disease [37]. Physical activity is thought to increase levels of anti-inflammatory cytokines [38]. Therefore, physical activity might have an inverse association with endometriosis. Accordingly, Ek et al. [18] found an inverse association between endometriosis and physical activity; an association which disappeared in the current study after adjustment for cofounders. A systematic review indicated an inverse association with physical activity but concluded that more studies are needed [38]. It should be considered that the inverse association between physical exercise and endometriosis might be due to that pain prevents the women from exercising.

Endometriosis is an estrogen-dependent disease [11], and previous studies have observed an association between estrogen-dependent diseases and alcohol [39]. Therefore, alcohol intake should be associated with endometriosis. Some studies have described such an association between endometriosis and alcohol intake [35, 40], whereas studies from our region point in the opposite direction [18]. The inverse association between endometriosis and alcohol consumption might not reflect causality.

Since the IBS diagnoses were self-reported, there could be a misreporting of the diagnosis from the participants. However, the significant association between self-reported IBS and IBS diagnosis from medical records suggests a small risk of misreporting. The present association between IBS and sick leave was a non-significant trend in the former, smaller MOS cohort [19]. The sensitivity analyses observed a positive association between IBS and smoking and an inverse association with age in line with previous research [19], although there are conflicting results among studies [41]. Other studies have also observed an association between abdominal and general pain and smoking [42, 43].

This population-based study did not observe any significant difference in the symptoms between women with endometriosis and IBS, which is in line with current research where women with endometriosis may fulfill the Rome criteria [9, 10, 17]. However, the symptoms were aggravated in IBS, especially pain, diarrhea, constipation, and bloating. The cohorts recruited from a tertiary center described symptom differences between the two entities [22], which underlines the importance of studying different cohorts. Both endometriosis and IBS were associated with sick leave, supporting the great impact of the diseases on quality of life [18, 44, 45]. When symptoms were related to endometriosis location, cyclic defecation pain, cyclic constipation, and longer stool evacuation time were more frequently found in rectal endometriosis compared to other locations [46]. Another study showed that deeply infiltrating endometriosis (DIE) in varying organs affected the symptoms, and DIE involving the bowel was associated with more frequent noncyclic pain and overall gastrointestinal pain [47]. Since the localization of endometriosis was not known in the current study, no such comparisons could be made.

This cross-sectional study from a population-based cohort might entail a more representative selection of people than a study enrolling participants with a specific diagnosis. Enrollment of a population-based cohort and a specific cohort from a tertiary center from the same region is a strength and made it possible to compare the same diseases at several levels. Other strengths of this survey are the use of validated questions about specific gastrointestinal symptoms and exclusion of drugs associated with IBS [26, 30]. However, there is a risk of selection bias with more well-educated and health-conscious participants in clinical studies, thus, not truly representing the general population. Furthermore. there is also a risk of recall bias with the selection of study participants to MOS from MDCS-CC. The main limitation of the present study was that it did not include participants with a diagnosis given at primary healthcare centers, which could possibly contribute to a small endometriosis group. A small group means higher risks of bias and inaccuracy in results. Furthermore, many participants did not answer the VAS-IBS. The information about endometriosis was taken from the National Patient Register, and did not include the mode of diagnosis, the localization and severity of the disease, or the treatment given. Thus, some of the endometriosis patients may not have been investigated by laparoscopy [3, 5], and the symptoms could not be adjusted for subgroups of endometriosis. Another weakness of the study was that the healthy comparison group did not exclude participants with other diseases that cause gastrointestinal symptoms, which caused higher VAS-IBS scores in the healthy group compared to the healthy female reference group without any diseases [27]. Assessing the gastrointestinal symptoms during the past 2 weeks is a limitation because of the risk of excluding symptoms from the past.

## Conclusion

In conclusion, endometriosis and IBS were associated. Whether the association is causal or depends on common risk factors or common pathogenesis could not be determined. No obvious difference in gastrointestinal symptoms was observed between the two conditions, thus making it difficult to clinically distinguish between the diagnoses from the gastrointestinal viewpoint. The question remains whether women with endometriosis are wrongly diagnosed with IBS or whether they suffer from both IBS and endometriosis. Additional and larger studies in this area are needed to differentiate between the diagnoses to be able to give the most optimal treatment to women with gastrointestinal symptoms.

## Data Availability

The datasets used and/or analyzed during the current study are available from the corresponding author on reasonable request.

## List of abbreviations

ATC-system: Anatomic therapeutic chemical classification system
BMI: Body mass index
CI: Confidence interval
DGBI: Disease of the gut-brain interaction
FASS: Farmaceutiska specialiteter i Sverige
FODMAP: Fermentable oligo-, di-, monosaccharides and polyols
IBS: Irritable bowel syndrome
MDCS-CC: Malmö Diet Cancer Study-Cardiovascular Cohort
MOS: Malmö Offspring Study
OR: Odds ratio
VAS-IBS: Visual analog scale for irritable bowel syndrome
WHO: World Health Organization

## References

[1] Bedaiwy MA, Alfaraj S, Yong P, Casper R (2017). New developments in the medical treatment of endometriosis. Fertil Steril.

[2] Brown J, Farquhar C (2014). Endometriosis: an overview of Cochrane Reviews. Cochrane Database Syst Rev.

[3] Nisenblat V, Bossuyt PM, Shaikh R, Farquhar C, Jordan V, Scheffers CS (2016). Blood biomarkers for the non-invasive diagnosis of endometriosis. Cochrane Database Syst Rev.

[4] Giudice LC, Kao LC, Endometriosis (2004). Lancet (Lond Engl).

[5] Hickey M, Ballard K, Farquhar C, Endometriosis (2014). BMJ.

[6] Oka P, Parr H, Barberio B, Black CJ, Savarino EV, Ford AC (2020). Global prevalence of irritable bowel syndrome according to Rome III or IV criteria: a systematic review and meta-analysis. Lancet Gastroenterol Hepatol.

[7] Shivaji UN, Ford AC (2014). Prevalence of functional gastrointestinal disorders among consecutive new patient referrals to a gastroenterology clinic. Frontline Gastroenterol.

[8] Lacy BE, Mearin F, Chang L, Chey WD, Lembo AJ, Simren M (2016). Bowel disorders. Gastroenterology.

[9] Chiaffarino F, Cipriani S, Ricci E, Mauri PA, Esposito G, Barretta M (2021). Endometriosis and irritable bowel syndrome: a systematic review and meta-analysis. Arch Gynecol Obstet.

[10] Nabi MY, Nauhria S, Reel M, Londono S, Vasireddi A, Elmiry M (2022). Endometriosis and irritable bowel syndrome: a systematic review and meta-analyses. Front Med (Lausanne).

[11] Nothnick W, Alali Z. Recent advances in the understanding of endometriosis: the role of inflammatory mediators in disease pathogenesis and treatment. F1000Res. 2016;5. 10.12688/f1000research.7504.1 PMC4760268 26949527

[12] Moore JS, Gibson PR, Perry RE, Burgell RE (2017). Endometriosis in patients with irritable bowel syndrome: specific symptomatic and demographic profile, and response to the low FODMAP diet. Aust N Z J Obstet Gynaecol.

[13] Eisenberg VH, Weil C, Chodick G, Shalev V (2018). Epidemiology of endometriosis: a large population-based database study from a healthcare provider with 2 million members. BJOG.

[14] Lovell RM, Ford AC (2012). Global prevalence of and risk factors for irritable bowel syndrome: a meta-analysis. Clin Gastroenterol Hepatol.

[15] Sibelli A, Chalder T, Everitt H, Workman P, Windgassen S, Moss-Morris (2016). R.A systematic review with meta-anaysis of the role of anxiety and depression in irritable bowel syndrome onset. Psychol Med.

[16] McGrath IM, Montgomery GW, Mortlock S. Insights from mendelian randomization and genetic correlation analyses into the relationship between endometriosis and its comorbidities. Hum Reprod Update. 2023:dmad009. 10.1093/humupd/dmad009 PMC10477944 37159502

[17] Saidi K, Sharma S, Ohlsson B (2020). A systematic review and meta-analysis of the associations between endometriosis and irritable bowel syndrome. Eur J Obstet Gynecol Reprod Biol.

[18] Ek M, Roth B, Nilsson PM, Ohlsson B (2018). Characteristics of endometriosis: a case-cohort study showing elevated IgG titers against the TSH receptor (TRAb) and mental comorbidity. Eur J Obstet Gynecol Reprod Biol.

[19] Nilsson D, Ohlsson B (2021). Gastrointestinal symptoms and irritable bowel syndrome are Associated with Female Sex and Smoking in the General Population and with unemployment in men. Front Med (Lausanne).

[20] Parasar P, Ozcan P, Terry KL, Endometriosis (2017). Epidemiology, diagnosis and clinical management. Curr Obstet Gynecol Rep.

[21] Cheong YC, Smotra G, Williams AC. Non-surgical interventions for the management of chronic pelvic pain. Cochrane Database Syst Rev. 2014(3):Cd008797. 10.1002/14651858.CD008797.pub2 PMC10981791 24595586

[22] Ek M, Roth B, Bengtsson M, Ohlsson B (2021). Gastrointestinal symptoms in women with endometriosis and microscopic colitis in comparison to irritable bowel syndrome: a cross-sectional study. Turk J Gastroenterol.

[23] Ek M, Roth B, Ekström P, Valentin L, Bengtsson M, Ohlsson B (2015). Gastrointestinal symptoms among endometriosis patients–A case-cohort study. BMC Womens Health.

[24] Brunkwall L, Jonsson D, Ericson U, Hellstrand S, Kennback C, Ostling G (2021). The Malmo offspring study (MOS): design, methods and first results. Eur J Epidemiol.

[25] Longstreth GF, Thompson WG, Chey WD, Houghton LA, Mearin F, Spiller RC (2006). Functional bowel disorders. Gastroenterology.

[26] Bengtsson M, Ohlsson B, Ulander K (2007). Development and psychometric testing of the Visual Analogue Scale for irritable bowel syndrome (VAS-IBS). BMC Gastroenterol.

[27] Bengtsson M, Hammar O, Mandl T, Ohlsson B (2011). Evaluation of gastrointestinal symptoms in different patient groups using the visual analogue scale for irritable bowel syndrome (VAS-IBS). BMC Gastroenterol.

[28] Obesity (2000). Preventing and managing the global epidemic. Report of a WHO consultation. World Health Organ Tech Rep Ser.

[29] WHO collaborating center for drug ststistics methodology. ATC/DDD Index 2022 [Internet]. Norwegian institute of public helath; 2021 [updated 2021-12-14; cited 2022-10-24. Available from: https://www.whocc.no/atc_ddd_index/.

[30] Ruderstam H, Ohlsson B. Self-reported IBS and gastrointestinal symptoms in the general population are associated with asthma, drug consumption and a family history of gastrointestinal diseases. Scand J Gastroenterol. 2022;1–11. 10.1080/00365521.2022.2031281. 10.1080/00365521.2022.2031281 35104172

[31] Pugsley Z, Ballard K (2007). Management of endometriosis in general practice: the pathway to diagnosis. Br J Gen Pract.

[32] Wróbel M, Wielgoś M, Laudański P (2022). Diagnostic delay of endometriosis in adults and adolescence-current stage of knowledge. Adv Med Sci.

[33] Agarwal SK, Chapron C, Giudice LC, Laufer MR, Leyland N, Missmer SA (2019). Clinical diagnosis of endometriosis: a call to action. Am J Obstet Gynecol.

[34] Blichfeldt-Eckhardt MR, Ording H, Andersen C, Licht PB, Toft P (2014). Early visceral pain predicts chronic pain after laparoscopic cholecystectomy. Pain.

[35] Zhang Y, Ma NY (2021). Environmental risk factors for endometriosis: an Umbrella Review of a Meta-analysis of 354 observational studies with over 5 million populations. Front Med (Lausanne).

[36] Bravi F, Parazzini F, Cipriani S, Chiaffarino F, Ricci E, Chiantera V (2014). Tobacco smoking and risk of endometriosis: a systematic review and meta-analysis. BMJ Open.

[37] Machairiotis N, Vasilakaki S, Thomakos N (2021). Inflammatory Mediators and Pain in Endometriosis: a systematic review. Biomedicines.

[38] Bonocher CM, Montenegro ML, Rosa ESJC, Ferriani RA, Meola J (2014). Endometriosis and physical exercises: a systematic review. Reprod Biol Endocrinol.

[39] Singletary KW, Gapstur SM (2001). Alcohol and breast cancer: review of epidemiologic and experimental evidence and potential mechanisms. JAMA.

[40] Parazzini F, Cipriani S, Bravi F, Pelucchi C, Chiaffarino F, Ricci E (2013). A metaanalysis on alcohol consumption and risk of endometriosis. Am J Obstet Gynecol.

[41] Creed F (2019). Review article: the incidence and risk factors for irritable bowel syndrome in population-based studies. Aliment Pharmacol Ther.

[42] Pisinger C, Aadahl M, Toft U, Birke H, Zytphen-Adeler J, Jørgensen T (2011). The association between active and passive smoking and frequent pain in a general population. Eur J Pain.

[43] Zia JK, Lenhart A, Yang PL, Heitkemper MM, Baker J, Keefer L (2022). Risk factors for Abdominal Pain-Related Disorders of Gut-Brain Interaction in adults and children: a systematic review. Gastroenterology.

[44] Klem F, Wadhwa A, Prokop LJ, Sundt WJ, Farrugia G, Camilleri M (2017). Prevalence, risk factors, and outcomes of irritable bowel syndrome after infectious enteritis: a systematic review and Meta-analysis. Gastroenterology.

[45] Shah E, Rezaie A, Riddle M, Pimentel M (2014). Psychological disorders in gastrointestinal disease: epiphenomenon, cause or consequence?. Ann Gastroenterol.

[46] Roman H, Ness J, Suciu N, Bridoux V, Gourcerol G, Leroi AM (2012). Are digestive symptoms in women presenting with pelvic endometriosis specific to lesion localizations? A preliminary prospective study. Hum Reprod.

[47] Fauconnier A, Chapron C, Dubuisson JB, Vieira M, Dousset B, Breart G. Relation between pain symptoms. and the anatomic location of deep infiltrating endometriosis. Fertil Steril. 2002;78(4):719–26. 10.1016/s0015-0282(02)03331-9 12372446

